# Unveiling Dynamic Changes of Chemical Constituents in Raw and Processed Fuzi With Different Steaming Time Points Using Desorption Electrospray Ionization Mass Spectrometry Imaging Combined With Metabolomics

**DOI:** 10.3389/fphar.2022.842890

**Published:** 2022-03-10

**Authors:** Yue Liu, Xuexin Yang, Chao Zhou, Zhang Wang, Tingting Kuang, Jiayi Sun, Binjie Xu, Xianli Meng, Yi Zhang, Ce Tang

**Affiliations:** ^1^ State Key Laboratory of Southwestern Chinese Medicine Resources, Chengdu University of Traditional Chinese Medicine, Chengdu, China; ^2^ School of Ethnic Medicine, Chengdu University of Traditional Chinese Medicine, Chengdu, China; ^3^ Waters Technology (Beijing) Co., Ltd., Beijing, China; ^4^ Innovative Institute of Chinese Medicine and Pharmacy, Chengdu University of Traditional Chinese Medicine, Chengdu, China

**Keywords:** Fuzi, DESI-MSI, diterpenoid alkaloids, toxicity attenuation, processing

## Abstract

Fuzi is a famous toxic traditional herbal medicine, which has long been used for the treatment of various diseases in China and many other Asian countries because of its extraordinary pharmacological activities and high toxicity. Different processing methods to attenuate the toxicity of Fuzi are important for its safe clinical use. In this study, desorption electrospray ionization mass spectrometry imaging (DESI-MSI) with a metabolomics-combined multivariate statistical analysis approach was applied to investigate a series of *Aconitum* alkaloids and explore potential metabolic markers to understand the differences between raw and processed Fuzi with different steaming time points. Moreover, the selected metabolic markers were visualized by DESI-MSI, and six index alkaloids’ contents were determined through HPLC. The results indicated visible differences among raw and processed Fuzi with different steaming times, and 4.0 h is the proper time for toxicity attenuation and efficacy reservation. A total of 42 metabolic markers were identified to discriminate raw Fuzi and those steamed for 4.0 and 8.0 h, which were clearly visualized in DESI-MSI. The transformation from diester-diterpenoid alkaloids to monoester-diterpenoid alkaloids and then to non-esterified diterpene alkaloids through hydrolysis is the major toxicity attenuation process during steaming. DESI-MSI combined with metabolomics provides an efficient method to visualize the changeable rules and screen the metabolic markers of *Aconitum* alkaloids during steaming. The wide application of this technique could help identify markers and reveal the possible chemical transition mechanism in the “Paozhi” processes of Fuzi. It also provides an efficient and easy way to quality control and ensures the safety of Fuzi and other toxic traditional Chinese medicine.

## 1 Introduction

Fuzi, the lateral root of *Aconitum carmichaelii* Debeaux (Fam. Ranunculaceae), primarily cultivated in Jiangyou and Butuo in Sichuan, China, has been an indispensable herb in traditional Chinese medicine (TCM) for thousands of years (Chinese Pharmacopoeia Commission, 2020). Jiangyou Fuzi and Butuo Fuzi are not only well-known genuine medicinal materials but also important agricultural products of Sichuan Province. The initial record of Fuzi can be dated back to the Han Dynasty in the earliest Chinese material medical classic work “Shennong Bencao Jing,” wherein it is listed in the inferior category because of its highly toxic properties (Singhuber et al., 2009; Zhou et al., 2015). However, Fuzi also has potent efficacy to restore from collapse, reinforce fire and Yang, and dispel cold to relieve pain (Luo et al., 2019; Chinese Pharmacopoeia Commission, 2020). It is usually combined with other herbs in formulations such as Si Ni Tang, Shen Fu Tang, and Fuzi Lizhong Wan to treat cold-damp-type illness (Singhuber et al., 2009). Given its outstanding clinical effects, Fuzi is one of “The Four Pillars” of TCM, together with *Ginseng Radix et Rhizoma*, *Rehmanniae Radix*, and *Rhei Radix et Rhizoma* (Dong et al., 2020). Modern studies have indicated that Fuzi has cardiotonic, anti-inflammatory, analgesic, and anti-tumor activities, which can be utilized to heal heart failure, shock, and hypotension subsequent to acute myocardial infarction, coronary heart disease, rheumatic heart disease, rheumatoid arthritis, tumors, skin wounds, depression, and diarrhea (Li et al., 2012; Zhao et al., 2012; Wu et al., 2018; Chinese Pharmacopoeia Commission, 2020). However, the toxicity of Fuzi coexists with its pharmaceutical activities, and thousands of Fuzi poisoning cases have been reported worldwide (Yang et al., 2018; Lei et al., 2021). Therefore, Fuzi is only used internally after processing, and various processing methods have been established by which the highly toxic alkaloids were transformed to lowly toxic and non-toxic forms through processing. The studies of toxic components and their mechanisms and the proper methods to attenuate the toxicity and reserve efficacy are the primary concern of scholars.

**FIGURE 1:**
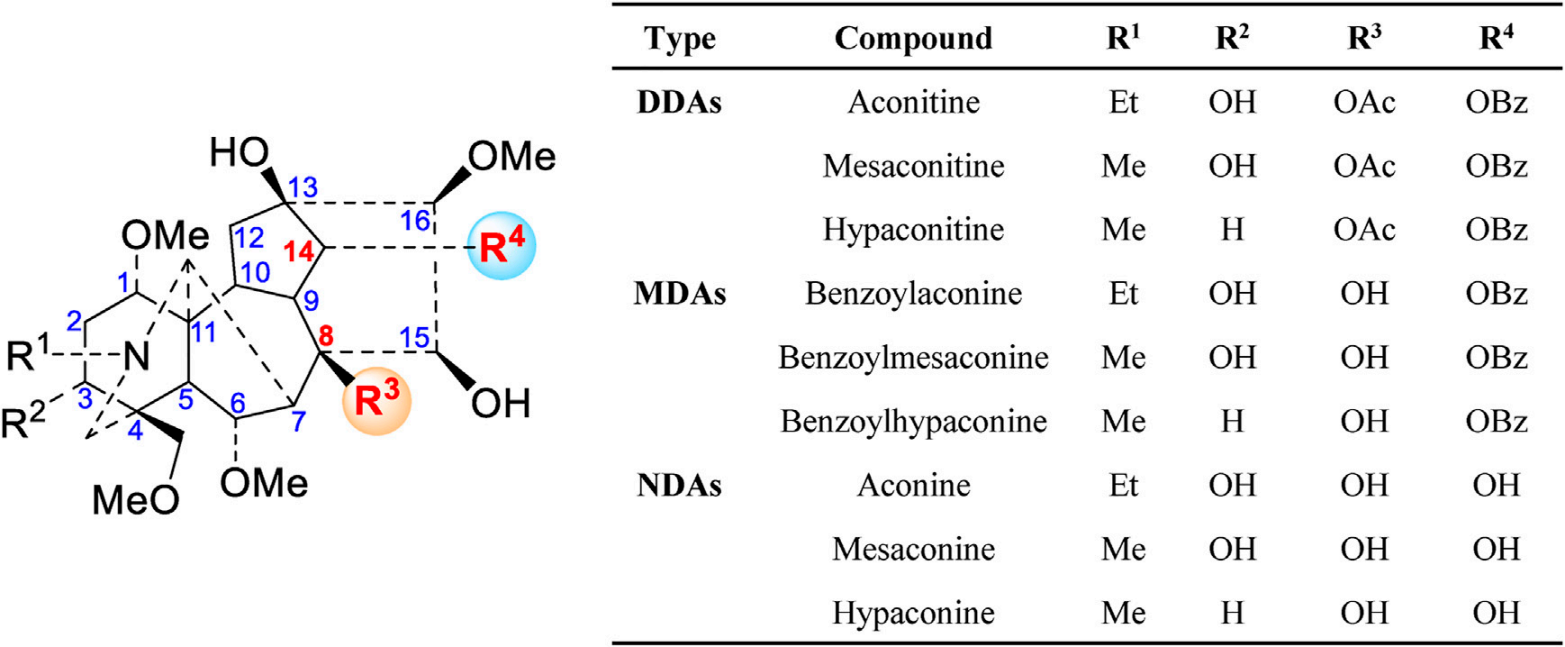
Main C_19_-diterpenoid alkaloids of Fuzi.

A literature survey has revealed that over 100 alkaloids have been isolated and identified in this herb, including C_20_-, C_19_-, and C_18_-diterpenoid alkaloids and double diterpenoid alkaloids, which are the main source of biological activities and toxicities. Among these alkaloids, the aconitine type, which belongs to C_19_-diterpenoid alkaloids, can be further divided into four categories: diester-diterpenoid alkaloids (DDAs), monoester-diterpenoid alkaloids (MDAs), non-esterified diterpene alkaloids (NDAs) (Figure 1), and lipo-diterpenoid alkaloids (LDAs). In recent years, numerous studies have demonstrated that the high toxicity of Fuzi is primarily attributed to DDAs (Tong et al., 2013). Based on previous studies, the main toxic effect of Fuzi is that it can affect the central nervous system, heart, and muscle tissues because of the interaction with voltage-dependent sodium channels, modulation of neurotransmitter release and related receptors, promotion of lipid peroxidation, and induction of cell apoptosis in the heart, liver, or other organs (Fu et al., 2006; Zhou et al., 2015; Liu et al., 2017). Therefore, the pretreatment method to reduce such toxic components is highly essential for the safe use of Fuzi.

The appropriate processing methods, which are known as “Paozhi” in Chinese, for TCM are usually necessary to reduce the toxicity while retaining or enhancing its pharmacological activities. Various methods can be used to process Fuzi, such as soaking, saturating, steaming, and decocting. According to the Chinese Pharmacopeia (CP) (2020 Edition), soaking in Danba (halogen solution), then continuous soaking with salt, steaming or decocting, long rinsing, and oven drying are the major processing methods for Fuzi (Chinese Pharmacopoeia Commission, 2020). During these heating procedures, the highly toxic DDAs were hydrolyzed to low toxic MDAs and non-toxic NDAs (Wang et al., 2003; Zhao et al., 2010; Tan et al., 2016). However, toxic *Aconitum* alkaloids are also responsible for the pharmacological activity and therapeutic efficacy of Fuzi, and efficacy preservation should be observed during toxicity attenuation. Recent research has indicated that DDAs, MDAs, and water-soluble active constituents have a massive loss after soaking in Danba and subsequent rinsing. Moreover, inorganic impurities are easily introduced to Fuzi (Ye et al., 2019). Thus, a Danba-free process becomes a more recognized processing approach for Fuzi. Nevertheless, the visual changes and distribution of DDAs and MDAs during steaming are rarely investigated. Therefore, a suitable methodology to explore these variation characteristics is highly necessary.

At present, the high-performance liquid chromatography (HPLC) and liquid chromatography coupled with mass spectrometry (LC-MS) are commonly used methods for alkaloid analysis of Fuzi (Yue et al., 2009; Xu et al., 2014; Yang et al., 2014; Miao et al., 2019; Sun et al., 2020). However, these techniques require complex preparation procedures, especially extraction from crushed samples. The ambient ionization MS, such as direct analysis in real-time mass spectrometry (DART-MS), was also utilized for direct analysis of Fuzi and other processed herbal medicine. Nevertheless, this method still needs sample pretreatment (Zhu et al., 2012). Therefore, analyzing and recognizing the distribution of specific alkaloids in original Fuzi has become challenging.

Mass spectrometry imaging (MSI) integrates sensitivities and high-throughput screening mass spectrometry with spatial and temporal chemical information and enables the visualized distribution of specific metabolites within non-destructive tissues (Stoeckli et al., 2001; Garza et al., 2018; Parrot et al., 2018). Matrix-assisted laser desorption/ionization (MALDI) and desorption electrospray ionization (DESI) are the commonly used ionization techniques for MSI (Parrot et al., 2018; Xu et al., 2019). Compared with MALDI-MSI, DESI-MSI allows 2D polar biomolecular distribution analysis with minimal sample pretreatment. Furthermore, no matrix is needed at ambient conditions (Takáts et al., 2004).

 The extensive application of DESI-MSI to determine natural products (primary and secondary metabolites) demonstrated its power and good practicability (Dill et al., 2009; Li et al., 2012; Hemalatha and Pradeep, 2013; Hemalatha et al., 2015; Liao et al., 2019; Mohana Kumara et al., 2019).

In this study, an easy-handling and comprehensive method combining DESI-MSI with metabolomics was applied to investigate the variation of key ester-diterpenoid alkaloids and identify the metabolic markers in raw and processed Fuzi with different steaming time points. In addition, the contents of six ester-type alkaloids were determined through HPLC.

## 2 Materials and Methods

### 2.1 Materials and Reagents

The reference standards (>98%, quantification grade) of aconitine (**40**), mesaconitine (**22**), hypaconitine (**36**), benzoylaconine (**34**), benzoylmesaconine (**32**), and benzoylhypaconine (**29**) were obtained from Chengdu Herbpurify Co., Ltd. (Chengdu, China). LC-MS-grade methanol (MeOH), formic acid (FA), acetonitrile, and isopropanol were purchased from Sigma-Aldrich (Sigma-Aldrich, United States). Ammonium acetate of HPLC grade was obtained from Chengdu Chron Chemical Co., Ltd. (Chengdu, China). The Elga Labwater Purelab system (Elga-Veolia, High Wycombe, United Kingdom) was used to obtain purified water for UPLC analyses. All other reagents were of analytical grade.

**FIGURE 2:**
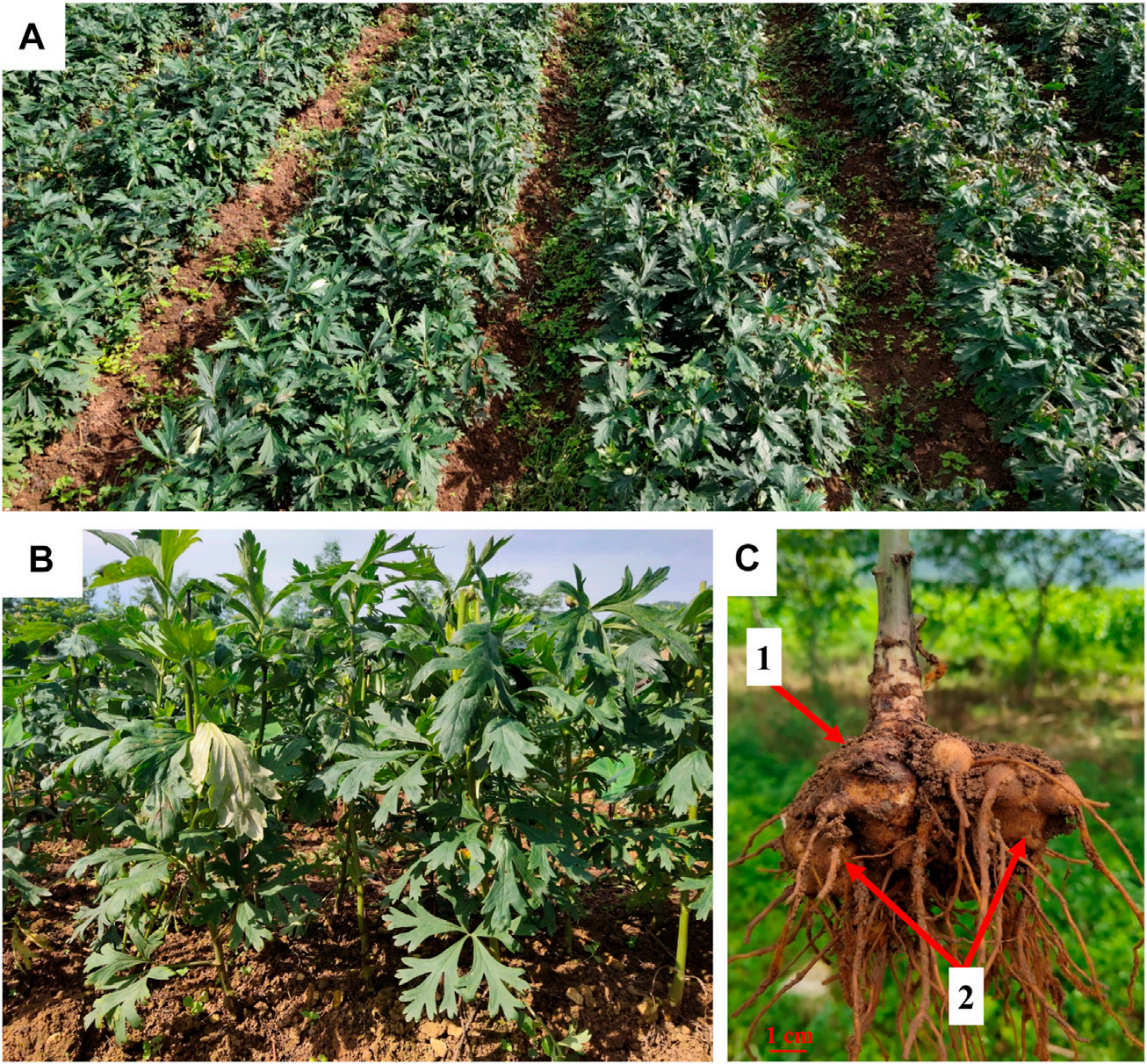
Fuzi planting base in Jiangyou, Sichuan **(A,B)**. The mother root (1) and lateral root (2) of *A. carmichaelii* **(C)**.

A batch of crude Fuzi was collected from Jiangyou (Sichuan, China) and authenticated by Professor Yi Zhang from Chengdu University of TCM (Figure 2). The voucher specimens were deposited in the School of Ethnic Medicine, Chengdu University of TCM, China.

### 2.2 Sample Preparation and DESI-MSI Analysis

Cleaned raw Fuzi were randomly divided into 11 groups, one of which was selected as raw Fuzi (without steaming), and the rest were placed into a suitable container for steaming for 1.0, 2.0, 3.0, 4.0, 5.0, 6.0, 7.0, 8.0, 9.0, and 10.0 h. All the samples were directly sliced, and the thickness of cross-sectional slices was approximately 5 mm. Subsequently, the sample was glued onto glass slides using double-sided tape and then subjected to DESI-MSI analysis.

Imaging experiments were conducted using a Waters Xevo G2-XS QTof mass spectrometer equipped with a DESI source (Waters Corporation, Milford, MA, United States). The following DESI parameters were optimized to obtain good signal intensity: nebulizing gas (dry nitrogen) pressure: 0.45 MPa; spray solvent 70% MeOH, 30% H_2_O, 0.2% FA, and 0.1 mM leucine enkephalin (LE) delivered at 2 μl/min; capillary voltage: 4.5 kV; and ionization mode: positive. The pixel size (X and Y pixel size of 200 μm) was determined on the basis of the total scan time of the mass spectra and the x–y scanner speed. A mass range of *m/z* 100–1,000 and the scan rate of 400 μm/s were used for imaging, and the images were viewed using high-definition image (HDI) v1.5 software (Waters Corporation, Milford, MA, United States). The MS images were created from Raw MS files through HDI with LE as the lockmass (m/z 556.2766) for resolution MS.

### 2.3 Desorption Electrospray Ionization Mass Spectrometry Imaging Data Processing and Statistical Analysis

MS raw data files were imported into HDI for imaging. The regions of interest (ROIs) were selected from root areas. Each sample had six ROIs that were converted into a data matrix of mass *m/z* and signal intensity. The data matrix was imported into SIMCA-P 13.0 (Umetrics, Umea, Sweden) to establish PAC, PLS-DA, and OPLS-DA models for distinguishing classification analysis and selecting crucial variable constituents for Fuzi steaming for different time points. The data were standardized using a Pareto-scaling technique.

### 2.4 Sample Preparation and HPLC Analysis

According to the CP (2020 Edition) (Chinese Pharmacopoeia Commission, 2020), all the processed or raw samples were oven-dried at 50°C for 24 h. Then, the dried samples were finely powered and passed through a 50-mesh sieve. Each sample powder (2.0 g) was weighed and then extracted by ultrasonication with 3 ml of ammonia TS and 50 ml of isopropanol–ethyl acetate (1/1, *v/v*) for 30 min (300 w, 40 kHz). Following extraction, isopropanol–ethyl acetate (1/1, *v/v*) was added to compensate for the lost weight during extraction and then filtered. 25 ml of filtrate was accurately measured and evaporated under reduced pressure at 40°C to dryness. Three milliliters of 0.05 M hydrochloric acid–methanol solution was precisely added to dissolve the residue. The supernatants were used as the sample solution after being centrifuged at 15,000 rpm for 30 min. The concentration of aconitine (AC, **40**), mesaconitine (MAC, **22**), hypaconitine (HAC, **36**), benzoylaconine (BAC, **34**), benzoylmesaconine (BMAC, **32**), and benzoylhypaconine (BHAC, **29**) in every sample was calculated on the basis of established calibration curves.

The filtered sample was analyzed using Agilent Technology 1260 Infinity (Agilent Technologies, Santa Clara, CA, United States) equipped with a binary pump, mobile phase degasser, temperature-controlled autosampler, column thermostat, and diode array detector (1260-DAD). HPLC separation was performed on an Agilent Eclipse XDB C_18_ column (4.6 mm × 250 mm, 5 μm) and maintained at 30°C. The mobile phase consisted of 0.04 M of ammonium acetate solution (solvent A, adjusting pH to 8.5 with an ammonia solution) and acetonitrile (solvent B) using the following gradient: 20%–28% B for 0–10 min, 28%–30% B for 10–20 min, 30%–35% B for 20–50 min, and 35%–38% B for 50–60 min. The flow rate was kept at 1 ml/min, and the injection volume was 10 μl. The UV detection wavelength was set at 235 nm.

HPLC-DAD was validated with regard to linearity, stability, recovery, repeatability, and precision. The storage solutions of six compounds were used in the regression equations between the peak areas and concentrations of six compounds. The limit of detection (LOD) and quantification (LOQ) were determined at signal-to-noise ratios of 3 and 10, respectively.

## 3 Results

### 3.1 Optimization of Spray Solvent for Desorption Electrospray Ionization Mass Spectrometry Imaging Analysis

**FIGURE 3:**
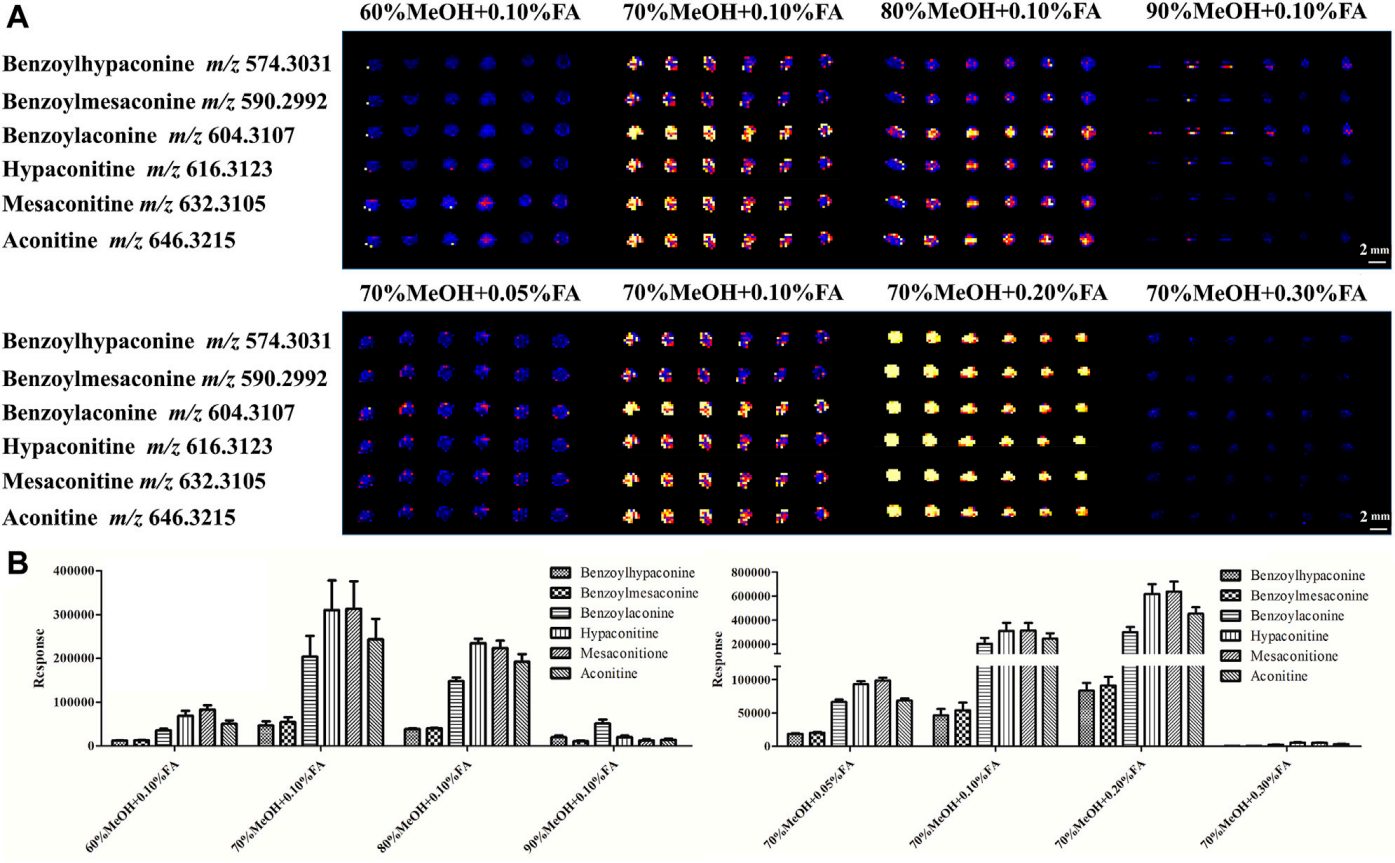
DESI-MS images **(A)** and responses **(B)** of six alkaloids using different concentrations of MeOH with different proportions of FA.

In DESI analysis, the ratio selection of solvent and acid is an important step to achieve sensitive and high MS signal response to target compounds. The solvent compositions are selected based on the analyte of interest and the tissue surface, which play a major role in the elution of each category of the compound (Claude et al., 2017). A large number of literature studies indicate that Fuzi primarily contains alkaloids. Therefore, a mixture of MeOH and water (with optional FA for positive ion mode analysis) is used as the DESI spray solvent for alkaloid analysis (Kumara et al., 2016). In the present experiment, the volume ratio of water to MeOH and FA concentration were evaluated (Supplementary Figure S1). As shown in Figures 3A,B, using 70% MeOH with 0.2% FA provided high response and uniform detection of six ester-type alkaloids across the Fuzi sample. Therefore, this solvent condition was used in the following MSI experiments.

### 3.2 Effects of Steaming on Toxicity and Active Components of Fuzi

As a highly toxic medicinal material, improper use of Fuzi will seriously affect the safety of people. According to the CP (2020 edition), DDAs shall not exceed 0.020% with regard to the total amount of mesaconitine (MAC, **22**), hypaconitine (HAC, **36**), and aconitine (AC, **40**). In addition, the total amount of MDAs, including benzoylmesaconine (BMAC, **32**), benzoylaconine (BAC, **34**), and benzoylhypaconine (BHAC, **29**), should not be less than 0.010%.

The changes of the six aforementioned alkaloids in the samples of Fuzi steamed for different time points were analyzed by HPLC and DESI-MSI. The HPLC data (Table 1) showed that the content of three DDAs decreased under 0.020%, and MDAs increased above 0.010% in the investigated samples after 3.0 h of steaming. However, the MDA levels slightly declined to 0.096% after 4.0 h of steaming and then stabilized over 0.100% until 9.0 h of steaming. At 10.0 h of steaming, the total content of three MDAs reduced again. Therefore, the requirements stipulated by the CP can be met by steaming Fuzi for over 3.0 h. In addition, three DDAs of Fuzi could not be detected by HPLC (below the detection limit) after 5.0 h. The results of the methodological study are shown in Supplementary Tables S1, S2.

**TABLE 1:** Content determination of six ester-type alkaloids through HPLC (*n* = 3).

Steaming time (h)	Content of MDAs (mg/g)			Proportion of MDAs (%)	Content of DDAs (mg/g)			Proportion of DDAs (%)
	BMAC (**32**)	BAC (**34**)	BHAC (**29**)		MAC (**39**)	AC (**40**)	HAC (**36**)	
0	0.0478	0.0126	—	0.006	0.6694	0.7095	0.3830	0.176
1.0	0.6698	0.1659	0.3179	0.115	—	0.3385	—	0.034
2.0	0.6053	0.1851	0.6687	0.146	—	0.2664	—	0.027
3.0	0.4103	0.0664	0.6561	0.113	—	0.1627	—	0.016
4.0	0.3639	0.0951	0.5022	0.096	—	0.0064	—	0.006
5.0	0.4212	0.1551	0.5578	0.113	—	—	—	—
6.0	0.3412	0.0646	0.7582	0.116	—	—	—	—
7.0	0.4145	0.0770	0.7992	0.129	—	—	—	—
8.0	0.4912	0.0817	0.7918	0.137	—	—	—	—
9.0	0.2623	0.0282	0.8971	0.119	—	—	—	—
10.0	0.3112	0.1098	0.4433	0.086	—	—	—	—

**FIGURE 4:**
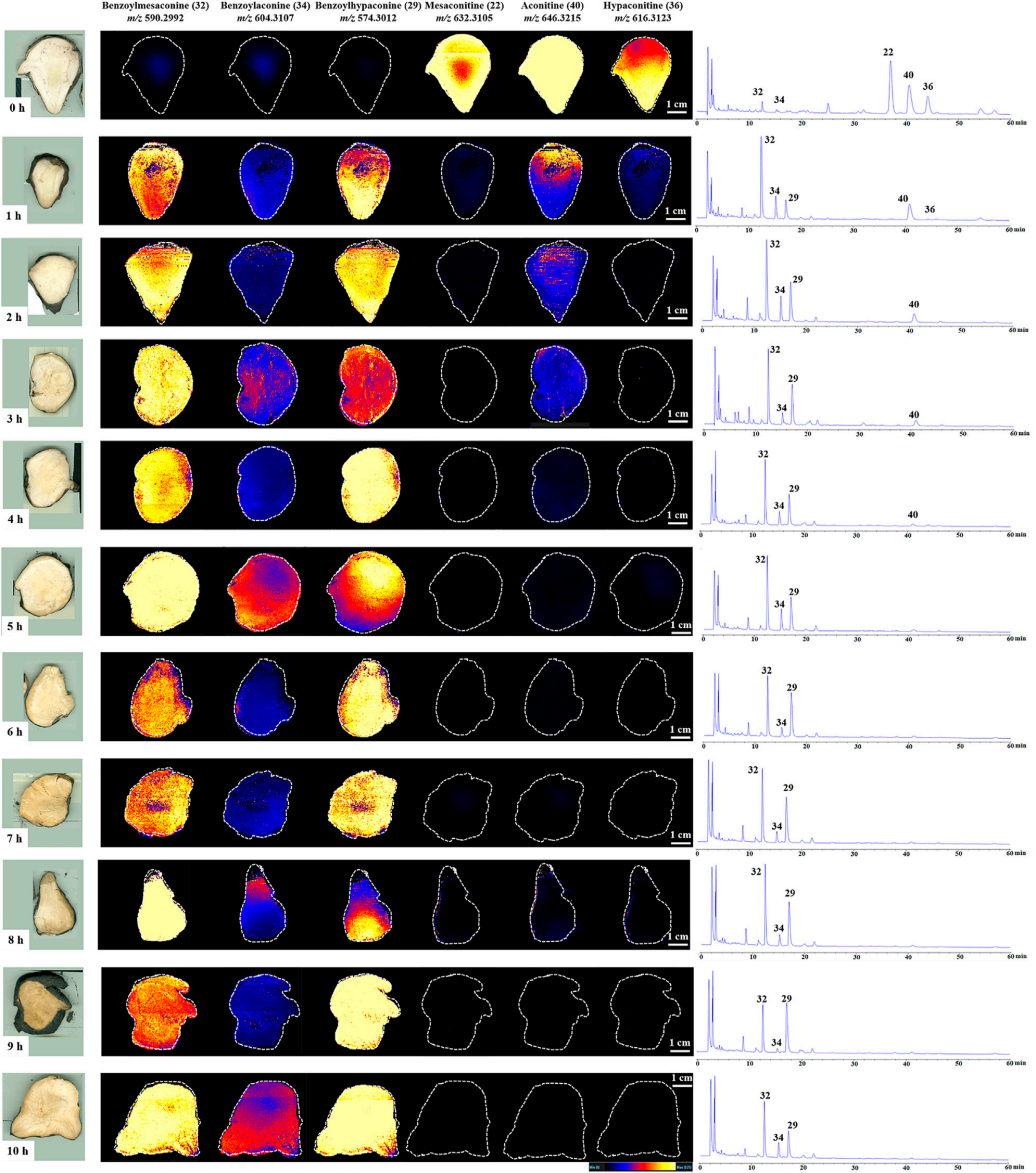
DESI-MS images and the corresponding HPLC chromatograms of six ester-type alkaloids in raw and processed Fuzi steamed for different time points.

*In situ* molecular detection was conducted in simulated industrial sections of Fuzi using DESI-MS with a mass range of m/z 100–1,000 in the positive ion mode to explore the change process of six alkaloids specified by the CP during different steaming time points of Fuzi. As shown in the MS images (Figure 4), six alkaloids, namely, mesaconitine (**22**, *m/z* 632.3105), aconitine (**40**, *m/z* 646.3215), hypaconitine (**36**, *m/z* 616.3123), benzoylmesaconine (**32**, *m/z* 590.2992), benzoylaconine (**34**, *m/z* 604.3107), and benzoylhypaconine (**29**, *m/z* 574.3012), were visually presented and compared. With the extension of steaming time, the concentrations of three DDAs decreased sharply, whereas the three MDAs increased significantly, among which mesaconitine (**22**) and hypaconitine (**36**) decreased faster than aconitine (**40**) and could not be visualized after 1.0 h, and the intensity of benzoylmesaconine (**32**) and benzoylhypaconine (**29**) increased faster than that of benzoylaconine (**34**). The DESI-MSI results are consistent with those of HPLC experiments, which indicated its reliability.

### 3.3 PCA and PLS-DA of Raw and Processed Fuzi With Different Steaming Hours

**FIGURE 5:**
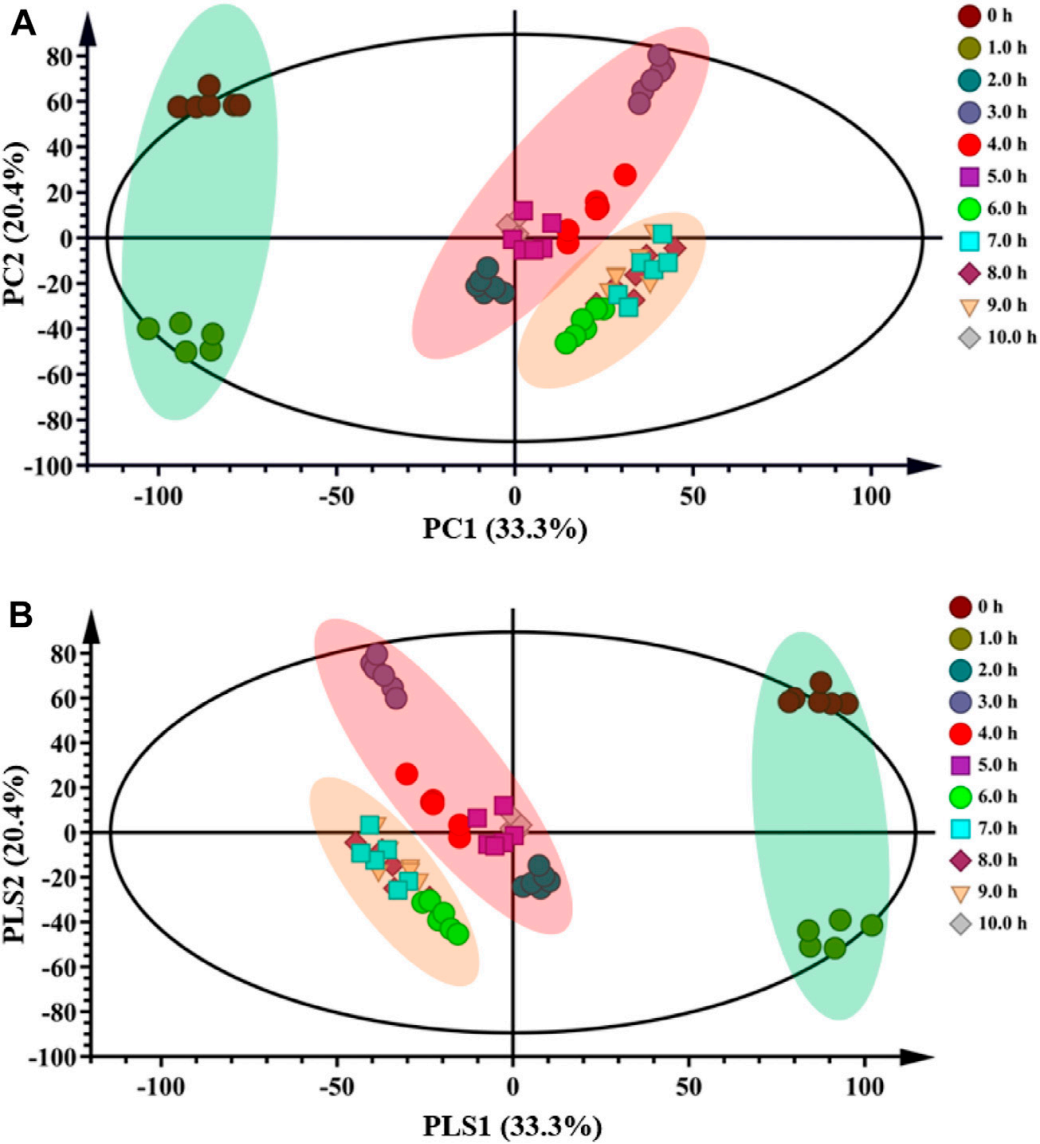
PCA **(A)** and PLS-DA **(B)** score plots of raw and processed Fuzi steamed for different time points.

A data matrix of mass *m*/*z* and signal intensity from DESI-MSI was submitted to PCA and PLS-DA to discriminate the variations of Fuzi with different steaming time points, visualize the underlying trend, and understand the metabolic differentiation among them. The PCA score plot (R^2^X = 0.884 and Q^2^ = 0.798) showed that all the ingredients could be classified into three major groups (Figure 5A). Raw Fuzi and processed Fuzi steamed only for 1.0 h were highly different from Fuzi steamed for more than 2.0 h. The ingredients of Fuzi steamed for 2.0–5.0 h were grouped together, but those steamed for 3.0 h were located relatively far away from the other samples. This may be due to the insufficient hydrolysis reaction from DDAs to MDAs. Fuzi steamed for 6.0–9.0 h were in a separate cluster. However, samples steamed for 10.0 h were back into the 2.0–5.0 h steaming group. The classification results also indicated that, after steaming for 5.0 h, these ingredients were located closer compared with those steamed for a short time. PLS-DA discrimination (R^2^X = 0.916, R^2^Y = 0.775, and Q^2^ = 0.701) (Figure 5B) was in good agreement with the PCA model, and all samples were separated into three groups. In addition, the samples steamed for a longer time were distributed closer. The close points indicated that these samples share similar chemical components. Combined with the results of HPLC, DESI-MSI, and multivariate statistical analysis, 4.0 h is demonstrated to be the appropriate steaming time for toxicity attenuation and efficacy reservation of Fuzi.

### 3.4 Metabolic Markers for the Identification of Raw and Processed Fuzi With Different Steaming Hours

**FIGURE 6:**
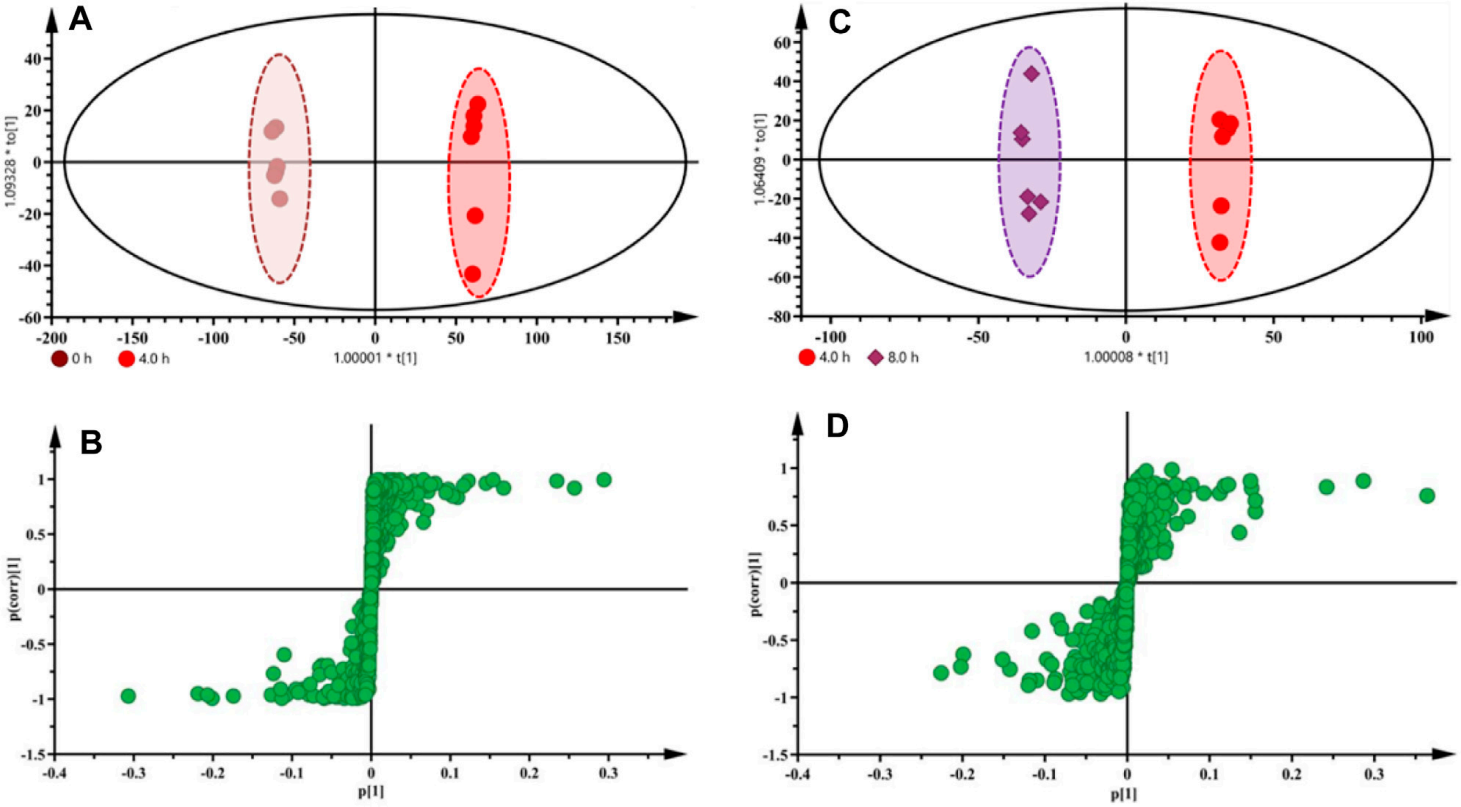
OPLS-DA score plots and S-plots based on raw and processed Fuzi steamed for 4.0 h **(A,B)** and Fuzi steamed for 4.0 and 8.0 h **(C,D)** from DESI-MSI data.

Based on the HPLC, DESI-MSI, PCA, and PLS-DA results, 4.0 and 8.0 h were selected as the key steaming time points to uncover the metabolic markers. OPLS-DA was applied to compare the metabolic changes between the 0 and 4.0 h groups and the 4.0 and 8.0 h groups. As shown in Figures 6A,C, the Fuzi samples with different steaming time points were separated from one another, indicating that the metabolic perturbation significantly occurred in raw Fuzi and processed Fuzi steamed at different time points. As shown in the corresponding S-plot (Figures 6B,D), the ions away from the origin contributed significantly to the separation between the 0 and 4.0 h groups and the 4.0 and 8.0 h groups. Therefore, the ions may be regarded as the discrimination metabolites for raw and processed Fuzi steamed for different hours. After combining the results of the S-plots, the VIP value (VIP >1) was obtained from OPLS-DA analysis, and the corresponding metabolites could be selected. The chemical structures of the metabolites were identified using online databases such as SciFinder (https://scifinder.cas.org/), and further confirmation was obtained through comparisons with authentic standards and published literature (Wang et al., 2002; Yue et al., 2007; Hu et al., 2009; Yan et al., 2010; Sun et al., 2012; Zhang et al., 2012; Sun et al., 2013; Xu et al., 2014; Yang et al., 2014; Luo et al., 2016; Zhang et al., 2017; Lei et al., 2021).

A total of 42 discriminating metabolites were tentatively identified, and the detailed information is given in Table 2 and Supplementary Figure S2. All 42 metabolites were visualized using DESI-MSI (Supplementary Figure S3). The intensities of 37 metabolites, including hypaconitine (**36**, *m/z* 616.3123), benzoylhypaconine (**29**, *m/z* 574.3012), neoline (**17**, *m/z* 438.2841), benzoylmesaconine (**32**, *m/z* 590.2992), mesaconitine (**39**, *m/z* 632.3105), aconitine (**40**, *m/z* 646.3215), and indaconitine (**38**, *m/z* 630.3278), changed after steaming for 4.0 h. Thus, these metabolites were considered as chemical markers to distinguish raw Fuzi and processed Fuzi steamed for 4.0 h. In addition, a total of 20 metabolites, such as neoline (**17**, *m/z* 438.2841), karacoline (**10**, *m/z* 378.2618), isotalatizidine/talatizidine (**13**, *m/z* 408.2755), benzoylhypaconine (**29**, *m/z* 574.3012), songorine (**6**, *m/z* 358.2386), fuzitine (**2**, *m/z* 342.1706), and talatisamine (**14**, *m/z* 422.2891), were set as the markers to distinguish Fuzi steamed for 4.0 and 8.0 h.

**TABLE 2:** Chemical information of 42 metabolic markers of Fuzi for different steaming time points.

No.	Putative identification	Molecular formula	[M+H]^+^		Delta (ppm)	MDa	VIP	
			Theoretical (*m/z*)	Measured (*m/z*)				
							VIP_0h/4h_	VIP_4h/8h_
**1**	Hetisine	C_20_H_27_NO_3_	330.2069	330.2090	6.4	2.1	3.29	—
**2**	Fuzitine	C_20_H_23_NO_4_	342.1705	342.1706	0.3	0.1	2.13	4.55
**3**	Denudatine/Guanfu base H	C_22_H_33_NO_2_	344.2590	344.2597	2	0.7	—	2.69
**4**	3-*epi*-Ignavinol	C_20_H_27_NO_4_	346.2018	346.2000	−5.2	−1.8	2.49	2.84
**5**	Dictysine	C_21_H_33_NO_3_	348.2539	348.2546	2	0.7	—	1.51
**6**	Songorine	C_22_H_31_NO_3_	358.2382	358.2386	1.1	0.4	3.06	6.65
**7**	Napelline	C_22_H_33_NO_3_	360.2539	360.2543	1.1	0.4	—	4.26
**8**	16-Hydroxycardiopetaline	C_21_H_33_NO_4_	364.2488	364.2479	−2.5	−0.9	1.37	3.58
**9**	Karakanine	C_22_H_33_NO_4_	376.2488	376.2495	1.9	0.7	1.22	1.51
**10**	Karacoline	C_22_H_35_NO_4_	378.2644	378.2618	−6.9	−2.6	2.54	8.23
**11**	Karacolidine/Chuanfumine	C_22_H_35_NO_5_	394.2593	394.2613	5.1	2	3.32	2.55
**12**	Nevadenine	C_23_H_35_NO_5_	406.2593	406.2585	−2	−0.8	—	1.86
**13**	Isotalatizidine/Talatizidine	C_23_H_37_NO_5_	408.2750	408.2755	1.2	0.5	5.29	7.22
**14**	Talatisamine	C_24_H_39_NO_5_	422.2906	422.2891	−3.6	−1.5	2.28	4.39
**15**	Senbusine A/B	C_23_H_37_NO_6_	424.2699	424.2707	1.9	0.8	2.68	—
**16**	Guiwuline	C_24_H_37_NO_6_	436.2699	436.2690	−2.1	−0.9	1.28	1.07
**17**	Neoline	C_24_H_39_NO_6_	438.2856	438.2841	−3.4	−1.5	8.17	11.09
**18**	Condelphine	C_25_H_39_NO_6_	450.2856	450.2849	−1.6	−0.7	1.05	—
**19**	Fuziline	C_24_H_39_NO_7_	454.2805	454.2823	4	1.8	3.79	—
**20**	14-*O*-acetyltalatizamine	C_26_H_41_NO_6_	464.3012	464.3033	4.5	2.1	1.45	—
**21**	Hypaconine	C_24_H_39_NO_8_	470.2754	470.2760	1.3	0.6	2.31	3.38
**22**	Mesaconine	C_24_H_39_NO_9_	486.2703	486.2706	0.6	0.3	1.87	3.32
**23**	8-Acetyl-15-hydroxyneoline	C_26_H_41_NO_8_	496.2910	496.2905	−1	−0.5	2.83	—
**24**	Episcopalisine	C_29_H_39_NO_6_	498.2856	498.2887	6.2	3.1	1.28	—
**25**	Aconicarchamine B	C_31_H_41_NO_7_	540.2961	540.2941	−3.7	−2	1.23	—
**26**	Dehydrated benzoylhypaconine	C_31_H_41_NO_8_	556.2910	556.2920	1.8	1	1.80	—
**27**	Straconitines A	C_31_H_43_NO_8_	558.3067	558.3067	0	0	1.14	—
**28**	6-*O*-benzoyleldelidine	C_32_H_43_NO_8_	570.3067	570.3087	3.5	2	1.63	—
**29**	Benzoylhypaconine	C_31_H_43_NO_9_	574.3016	574.3012	−0.7	−0.4	9.03	6.65
**30**	Pyraconitine	C_32_H_43_NO_9_	586.3016	586.3032	2.7	1.6	3.00	—
**31**	Benzoyldeoxyaconine	C_32_H_45_NO_9_	588.3173	588.3150	−3.9	−2.3	2.10	—
**32**	Benzoylmesaconine	C_31_H_43_NO_10_	590.2965	590.2992	4.6	2.7	7.24	—
**33**	*N*-demethylhypaconitine	C_32_H_43_NO_10_	602.2965	602.3012	7.8	4.7	1.24	—
**34**	Benzoylaconine	C_32_H_45_NO_10_	604.3122	604.3107	−2.5	−1.5	—	2.12
**35**	10-Hydroxybenzoylmesaconine	C_31_H_43_NO_11_	606.2914	606.2922	1.3	0.8	3.63	—
**36**	Hypaconitine	C_33_H_45_NO_10_	616.3122	616.3123	0.2	0.1	9.45	2.24
**37**	10-Hydroxybenzoylaconine	C_32_H_45_NO_11_	620.3071	620.3085	2.3	1.4	1.23	—
**38**	Indaconitine	C_34_H_47_NO_10_	630.3278	630.3278	0	0	6.17	—
**39**	Mesaconitine	C_33_H_45_NO_11_	632.3071	632.3105	5.4	3.4	6.85	—
**40**	Aconitine	C_34_H_47_NO_11_	646.3227	646.3215	−1.9	−1.2	6.37	—
**41**	Beiwutine	C_33_H_45_NO_12_	648.3020	648.3047	4.2	2.7	3.33	—
**42**	Aconifine	C_34_H_47_NO_12_	662.3177	662.3190	2	1.3	2.26	—

**FIGURE 7:**
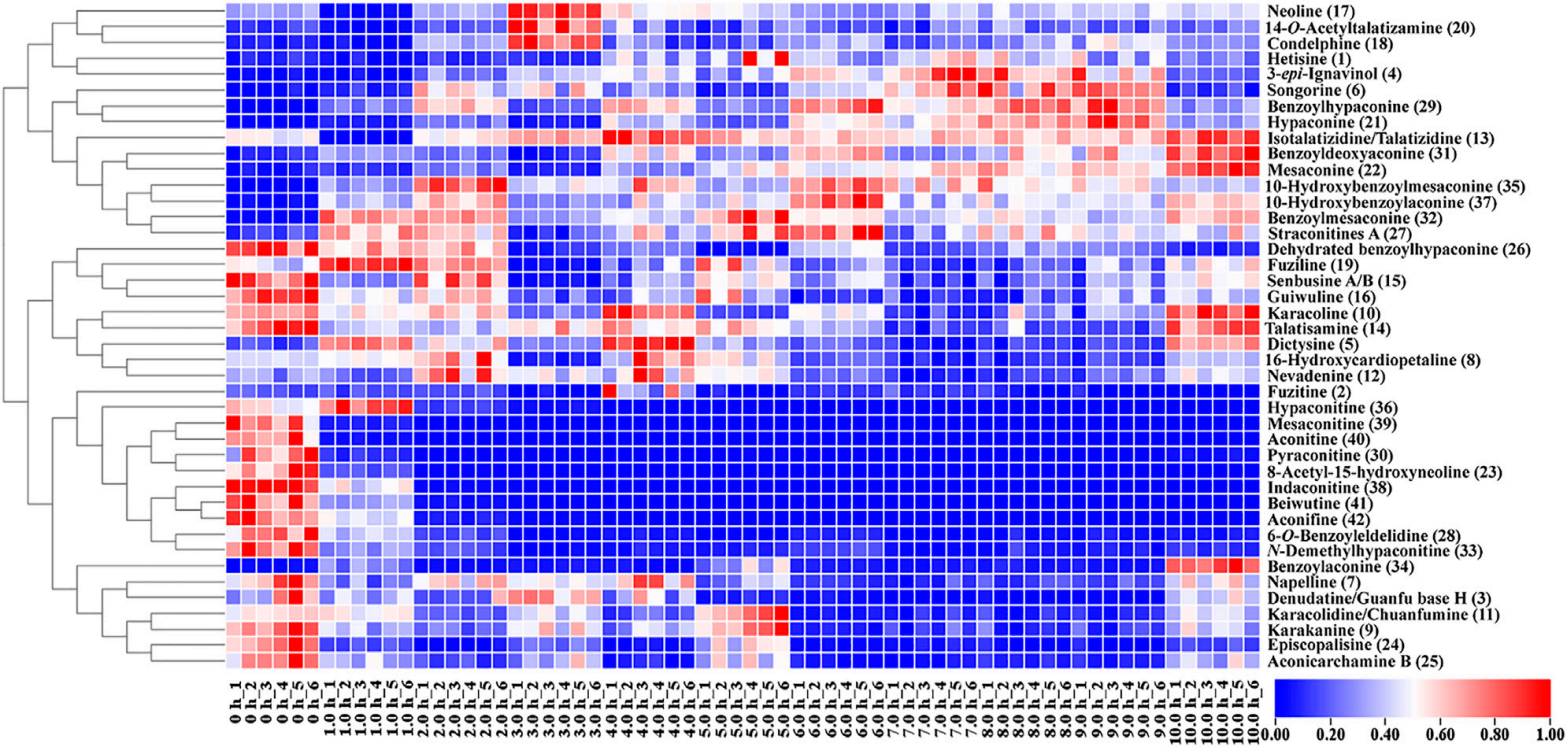
Heat map of metabolic markers of raw and processed Fuzi steamed for different time points.

A heat map was used to visualize the changes in the content of the 42 metabolites in raw Fuzi and processed Fuzi for different steaming hours. As shown in Figure 7, the content of 15 metabolites increased, whereas that of 27 metabolites decreased. Metabolites with increased content are primarily MDAs. However, the content of most DDAs, including three highly toxic ones, mesaconitine (**22**), aconitine (**40**), hypaconitine (**36**), and some of the MDAs decreased dramatically within two steaming hours. The content of napelline (**7**), denudatine/Guanfu base H (**3**), karakolidine/Chuanfumine (**11**), karakanine (**9**), episcopalisine (**24**), and aconicarchamine B (**25**) declined to low levels after steaming for 4.0–5.0 h, but such content increased again when steaming was continued for 10.0 h. Nine metabolites, including dehydrated benzoylhypaconine (**26**), fuziline (**19**), senbusine A/B (**15**), guiwuline (**16**), karacoline (**10**), talatisamine (**14**), dictysine (**5**), 16-hydroxycardiopetaline (**8**), and nevadenine (**12**), showed volatility decreases, and most of them belong to NDAs. As an isoquinoline alkaloid, fuzitine (**2**) generally exists in the form of salt, which showed a particular changing rule during steaming. The content reached the highest on steaming after 4.0 h and decreased or transformed to other metabolites with the extension of steaming time. The heat map also indicated that some of the metabolites were unstable when steamed for 3.0 h and then tended to be stable. When steamed for 10.0 h, the content of metabolites became similar to samples steamed for 4.0–5.0 h. This phenomenon corresponds to the aforementioned PCA and PLS-DA results (Figure 6).

## 4 Discussion

As a typical toxic traditional herbal medicine that is known for its extraordinary pharmacological activities and toxicity, Fuzi has long been used as an irreplaceable traditional herbal medicine in TCM, Kampo medicine, and homeopathy for thousands of years (Zhou et al., 2015; Wu et al., 2018; Qiu et al., 2021).

As toxicity and efficacy are interdependent in Fuzi, the toxicities of processed products such as Yanfuzi, Heishunpian, Baifupian, Danfupian, and Paofupian dramatically decreased compared with raw Fuzi (Zhou et al., 2012; Zhao et al., 2014), but the efficacy also decreased. Considering that Danba is needed during the aforementioned procedures, which would induce halide ions and inorganic impurities into Fuzi, the alkaloids will be lost with a long rinsing time (Ye et al., 2019). Recent studies indicated that the total alkaloids and various ester-diterpenoid alkaloids’ levels were high in directly steamed, stir-fried, or baked Fuzi, which showed better detoxification and efficacy reservation effects than Danba-used procedures (Liu et al., 2019; Ye et al., 2019). Thus, this study focused on direct steaming, discussed changeable rules of the key ester-diterpenoid alkaloids, and identified the metabolic markers in raw and processed Fuzi steamed at different time points through DESI-MSI combined with metabolomics.

Investigations of basal metabolism are important to improve the quality control of TCMs (Jiang et al., 2022). DDAs, the major pharmaceutical and toxic secondary metabolites, are important to determine the quality, safety, and efficacy of Fuzi, which were hydrolyzed during heating processing procedures, such as steaming. However, the changeable patterns during steaming are rarely reported. Here, DESI-MSI provides an easy and effective way to explore the change characteristics of the key ester-diterpenoid *Aconitum* alkaloids. Combining metabolism and multivariate statistical analysis, the metabolites and important markers would be identified, which are essential to assess the quality of steamed Fuzi.

HPLC was initially applied to determine variation in the content of three MDAs and three DDAs. A total of 10 steaming time points were set, and HPLC data indicated that the content limit of steamed Fuzi met the requirements of the CP after 3.0 h of steaming and became steady after 4.0 h. DESI-MSI was performed to visualize the HPLC results with high resolutions in mass and space: three MDAs were difficult to detect, and three DDAs were highly visible in raw Fuzi. The images showed an increase in MDAs and a decrease in DDAs during steaming. After 4.0 h of steaming, DDAs and MDAs met the standards of CP, and parts of the DDAs still remain. PCA and PLS-DA analyses also illustrated that the samples after steaming for over 4.0 h were grouped closer and located in the coordinate origin region. Consequently, 4.0 h of steaming was recommended for the toxicity attenuation and efficacy reservation of Fuzi.

**FIGURE 8:**
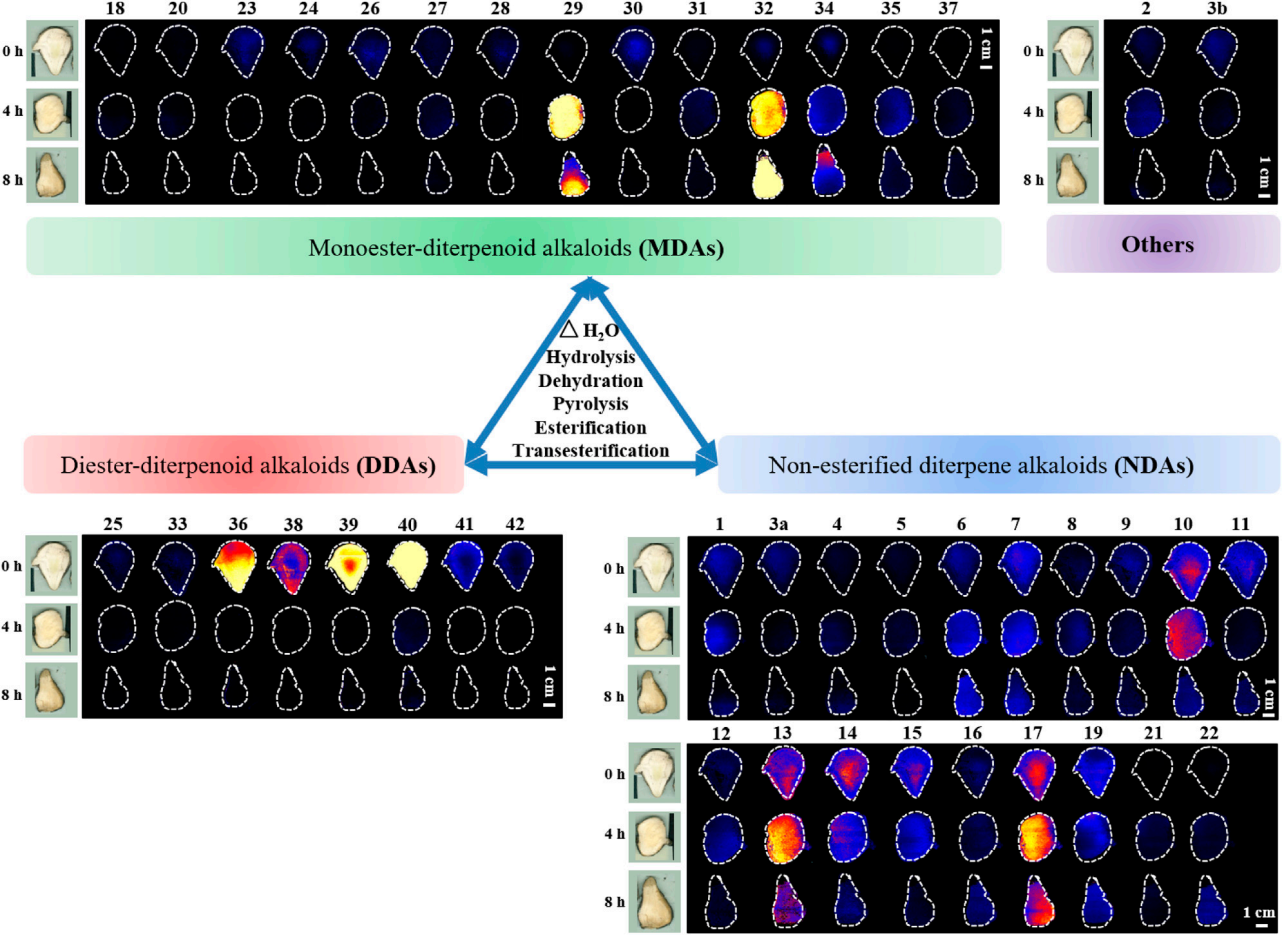
DESI-MS images of 42 metabolic markers in raw and processed Fuzi steamed for 4.0 and 8.0 h.

**FIGURE 9:**
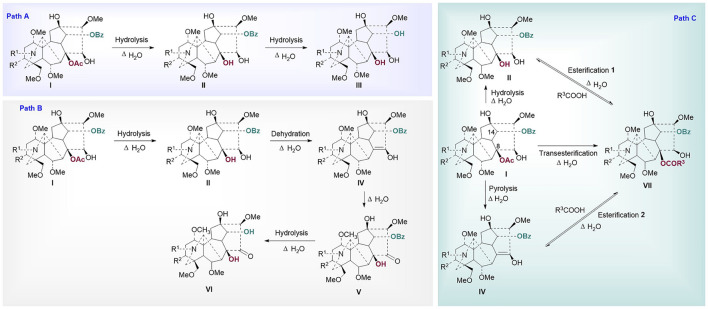
Proposed mechanism for the transformation of ester type alkaloids (Path **(A)**: hydrolysis reactions; Path **(B)**: hydrolysis and dehydration reactions; Path **(C)**: esterification and transesterification reactions I: diester-diterpenoid alkaloids, II: monoester-diterpenoid alkaloids; III: non-esterified diterpene alkaloids; IV: enol-type monoester-diterpenoid alkaloids; V: ketone-type monoester-diterpenoid alkaloids; VI: ketone-type non-esterified diterpene alkaloids; VII: lipo-diterpenoid alkaloids).

Chemometric analysis was combined with a heat map to identify the significant metabolic markers, and 4.0 h was set as the key time node. The DESI-MSI comparison of the 42 metabolites of raw Fuzi and processed Fuzi steamed for 4.0 and 8.0 h showed that the content of highly toxic DDAs decreased dramatically, and a large part of the content of MDAs and NDAs fluctuated during steaming, which indicated a reversible process (Figure 8). DDAs and MDAs were the major metabolic markers between raw and processed Fuzi steamed for 4.0 h, whereas NDAs were identified as the predominant markers of Fuzi steamed for 4.0 and 8.0 h. During 0–4.0 h of steaming, hydrolysis was conducted to convert DDAs to MDAs and then to NDAs, which are considered as the major chemical transformations based on the present results and published literature (Liu et al., 2008; Chen et al., 2013; Qiu et al., 2021), and three main paths for the transformation of C_19_-ester-diterpenoid alkaloids were proposed (Figure 9). Except for conventional hydrolysis and dehydration processes (Figures 9A,B), esterification reactions **1** and **2** in path C also indicated two important reversible processes, which explained why some of the alkaloids’ content fluctuated during steaming. However, NDAs were the important hydrolysis products after long and continuous steaming, and the pharmaceutical activities will then decrease.

## 5 Conclusion

In this study, the combination of DESI-MSI and metabolomics for rapid and high-resolution characterization of ester alkaloids in raw and steamed Fuzi was developed. The changes in alkaloids of raw and processed Fuzi with different steaming time periods were observed, and the results of HPLC, DESI-MS coupled with PCA, PLS-DA, and OPLS-DA all indicated that 4.0 h of steaming was appropriate for toxicity attenuation and efficacy reservation of Fuzi. In addition, a total of 37 metabolic markers to distinguish raw and processed Fuzi steamed for 4.0 h and 22 metabolites to distinguish Fuzi steamed for 4.0 and 8.0 h were identified through DESI-MSI-based metabolomics. Moreover, three major chemical transformation pathways of C_19_-ester alkaloids were summarized. Therefore, the novel method provides an efficient approach to visualize the changeable rules and screen the metabolic markers of alkaloids during steaming. The wide application of the method could help to identify biomarkers and reveal the possible chemical transition mechanism in the “Paozhi” processes of TCM.

## Data Availability

The raw data supporting the conclusion of this article will be made available by the authors, without undue reservation.
