# Supplementary material for: Unveiling Dynamic Changes of Chemical Constituents in Raw and Processed Fuzi With Different Steaming Time Points Using Desorption Electrospray Ionization Mass Spectrometry Imaging Combined With Metabolomics
Source: Front Pharmacol. 2022 Mar 10;13:842890. doi: 10.3389/fphar.2022.842890 (PMC8960191; doi:10.3389/fphar.2022.842890)
Supplement: Supplementary file 1 [file Table1.docx]

Supplementary Material

**Table S1.** HPLC-DAD detection parameters, regression equations, linear range, precision, repeatability, and stability of six ester-type alkaloids.

| **Compound** | **t_R_**  **(min)** | **Regression equation** | **r^2^** | **Linear range (μg/mL)** | **LOD** | **LOQ** | **Precision** | | **Repeatability** | **Stability** |
| --- | --- | --- | --- | --- | --- | --- | --- | --- | --- | --- |
|  |  |  |  |  |  |  | **Intraday**  **(n=6)** | **Interday**  **(n=3)** |  |  |
| **32** | 12.54 | *y* = 14.006*x* – 3.3303 | 0.9998 | 8.5~272 | 1.2 | 4.3 | 0.63 | 3.68 | 0.98 | 0.93 |
| **34** | 15.23 | *y* = 12.716*x* + 5.8969 | 0.9997 | 5.88~376 | 1.0 | 3.0 | 1.29 | 2.87 | 1.60 | 1.48 |
| **29** | 17.26 | *y* = 6.8904*x* – 7.5308 | 0.9994 | 9.5~304 | 1.5 | 4.8 | 1.82 | 2.29 | 2.07 | 1.94 |
| **22** | 37.32 | *y* = 15.892*x* – 8.7582 | 0.9996 | 12.25~392 | 1.4 | 6.1 | 1.51 | 1.58 | 2.22 | 2.15 |
| **40** | 41.07 | *y* = 10.959*x* – 1.9662 | 0.9996 | 9.5~304 | 1.4 | 4.8 | 0.76 | 1.10 | 0.97 | 0.82 |
| **36** | 44.46 | *y* = 10.478*x* – 5.8567 | 0.9996 | 12~384 | 1.5 | 6.0 | 1.93 | 2.04 | 2.42 | 2.38 |

Note: In the regression equation, *x* is the peak area, *y* the concentration of each analyte (µg/mL), and r the correlation coefficient. LOD, limit of detection (S/N=3), LOQ, limit of quantification (S/N=10). Intra-, inter-day precision, repeatability, and stability are shown in RSD (%).
