# Supplementary material for: Unveiling Dynamic Changes of Chemical Constituents in Raw and Processed Fuzi With Different Steaming Time Points Using Desorption Electrospray Ionization Mass Spectrometry Imaging Combined With Metabolomics
Source: Front Pharmacol. 2022 Mar 10;13:842890. doi: 10.3389/fphar.2022.842890 (PMC8960191; doi:10.3389/fphar.2022.842890)
Supplement: Supplementary file 2 [file Image2.pdf]

Supplementary Material

1 Supplementary Figures

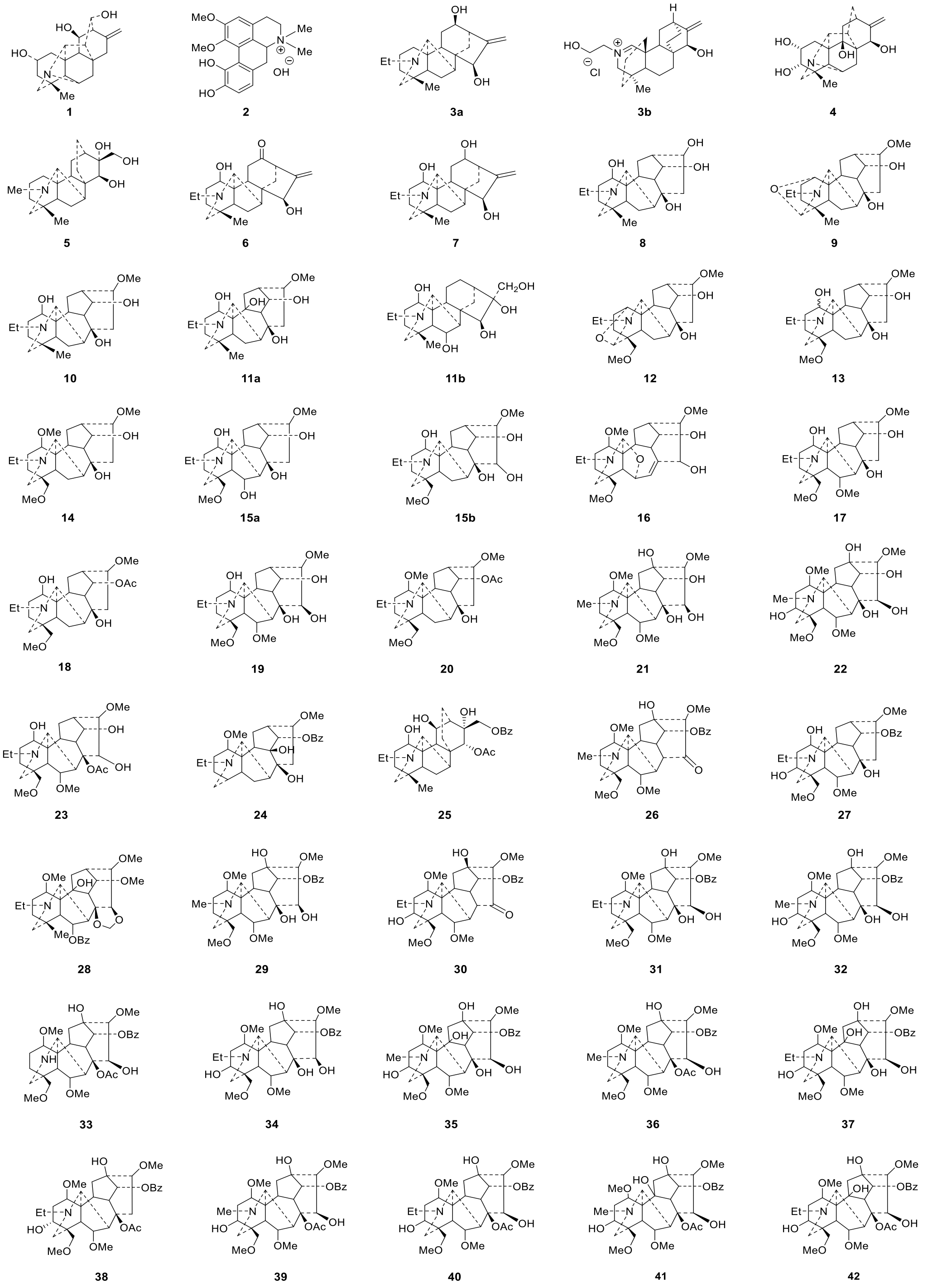

Supplementary Figure S2. Chemical structures of 42 metabolic markers of raw and processed Fuzi steamed for 0, 4.0 and 8.0 h.
