# Supplementary material for: Unveiling Dynamic Changes of Chemical Constituents in Raw and Processed Fuzi With Different Steaming Time Points Using Desorption Electrospray Ionization Mass Spectrometry Imaging Combined With Metabolomics
Source: Front Pharmacol. 2022 Mar 10;13:842890. doi: 10.3389/fphar.2022.842890 (PMC8960191; doi:10.3389/fphar.2022.842890)
Supplement: Supplementary file 3 [file Image3.pdf]

Supplementary Material

1 Supplementary Figures

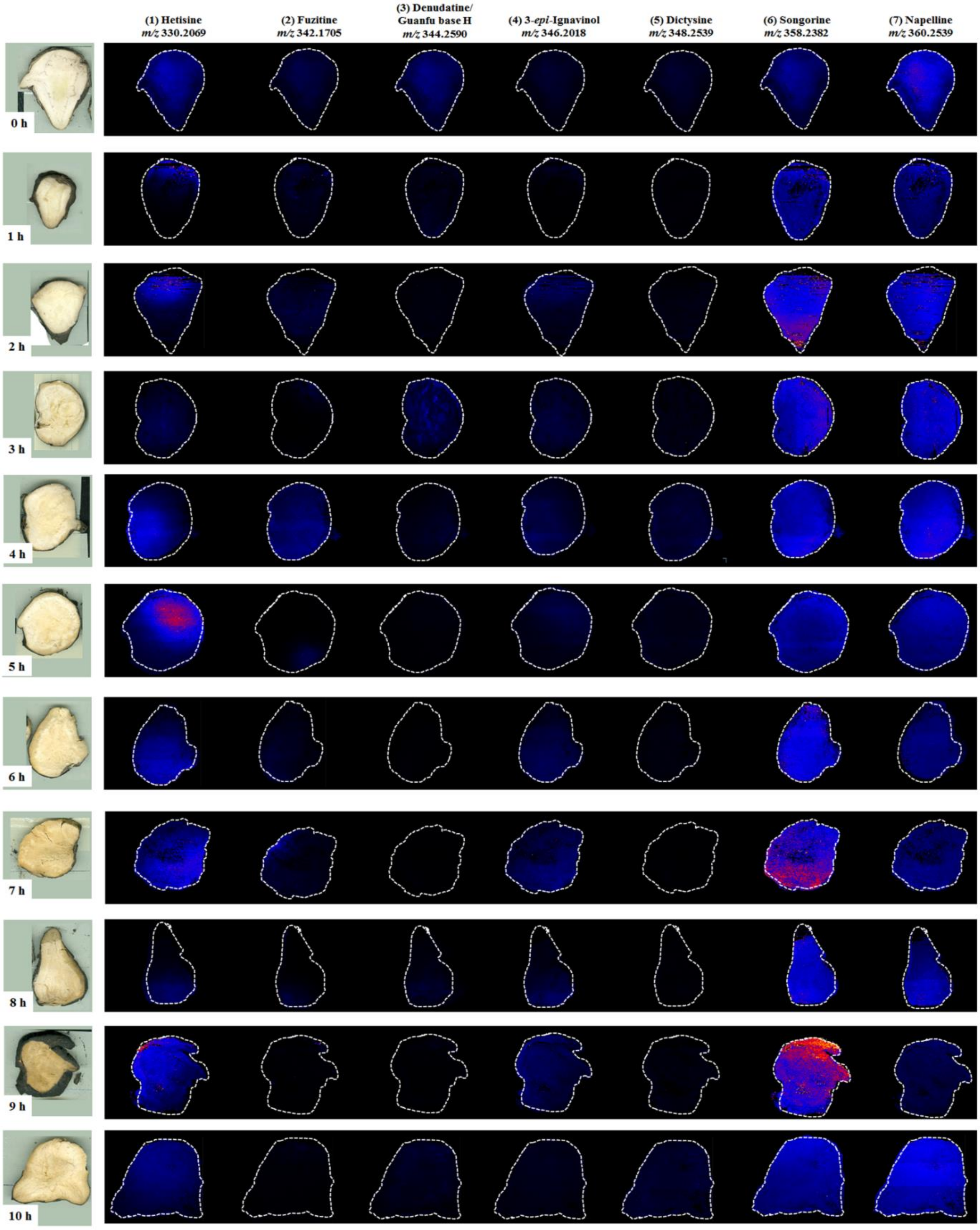

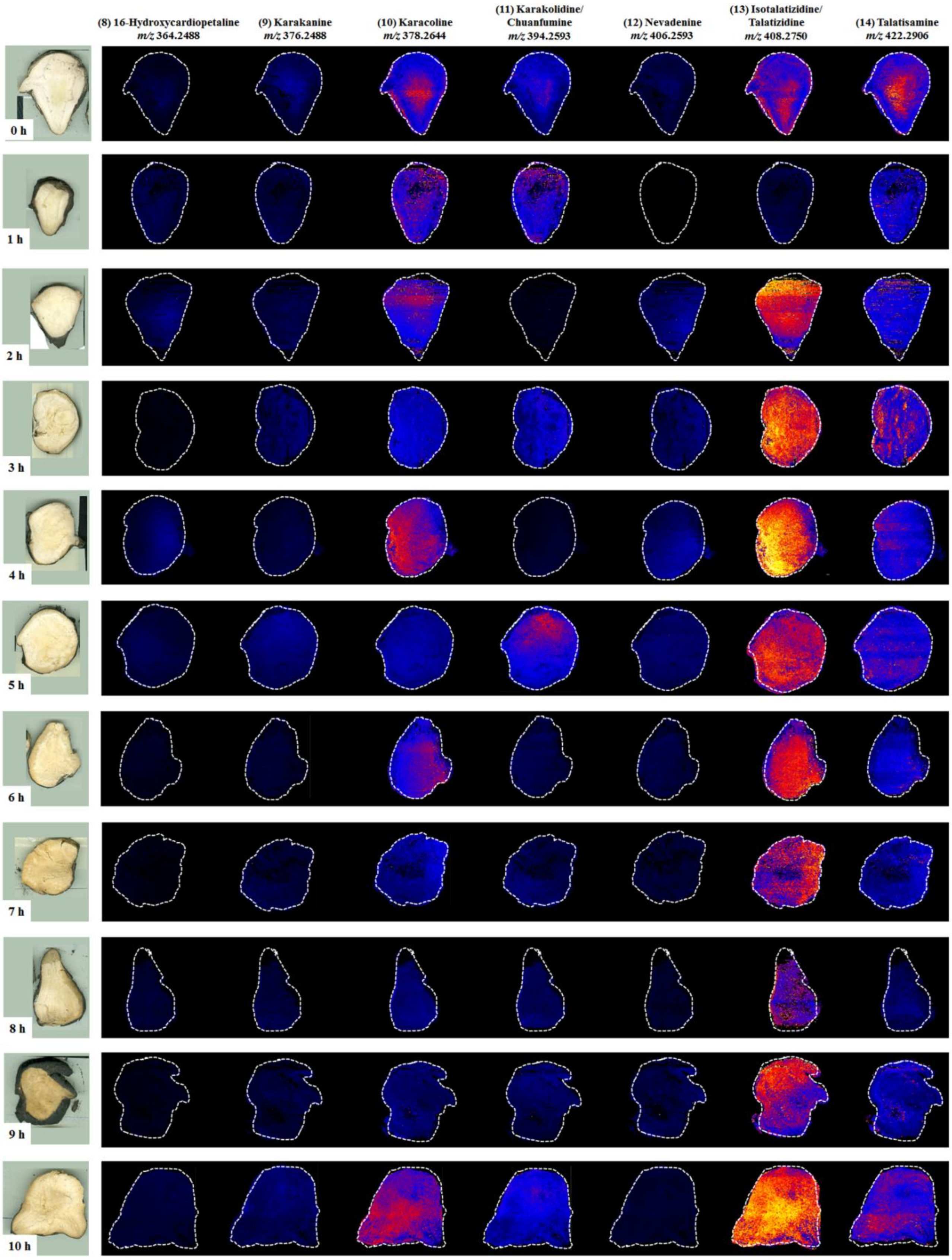
(8) 16-Hydroxycardiopetaline
m/z 364.2488
(9) Karakanine
m/z 376.2488
(10) Karacoline
m/z 378.2644
(11) Karakolidine/
Chuanfumine
m/z 394.2593
(12) Nevadenine
m/z 406.2593
(13) Isotalatizidine/
Talatizidine
m/z 408.2750
(14) Talatisamine
m/z 422.2906
0 h
1 h
2 h
3 h
4 h
5 h
6 h
7 h
8 h
9 h
10 h

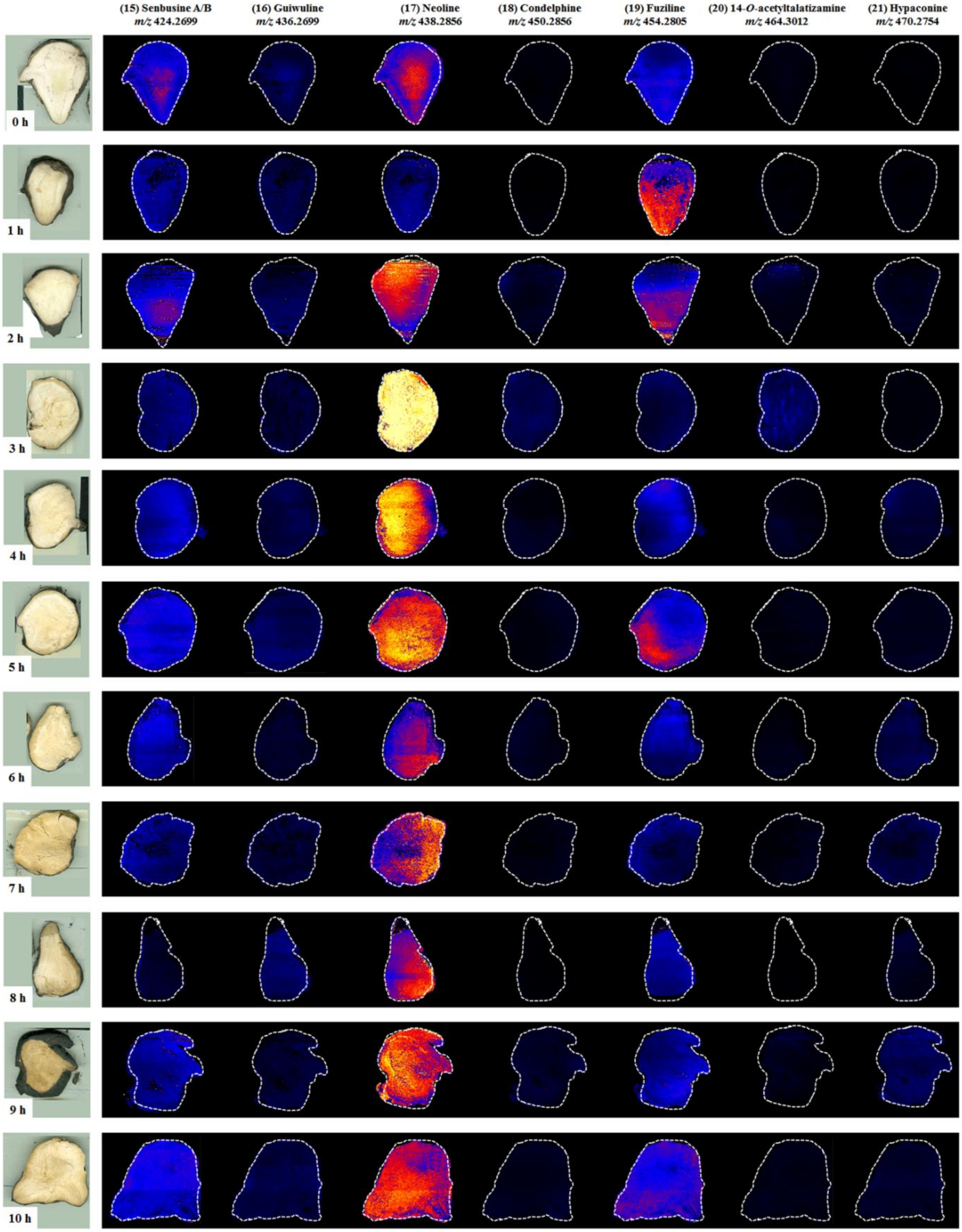

(15) Senbusine A/B
m/z 424.2699
(16) Guiwuline
m/z 436.2699
(17) Neoline
m/z 438.2856
(18) Condelphine
m/z 450.2856
(19) Fuziline
m/z 454.2805
(20) 14-O-acetyltalatizamine
m/z 464.3012
(21) Hypaconine
m/z 470.2754
0 h
1 h
2 h
3 h
4 h
5 h
6 h
7 h
8 h
9 h
10 h

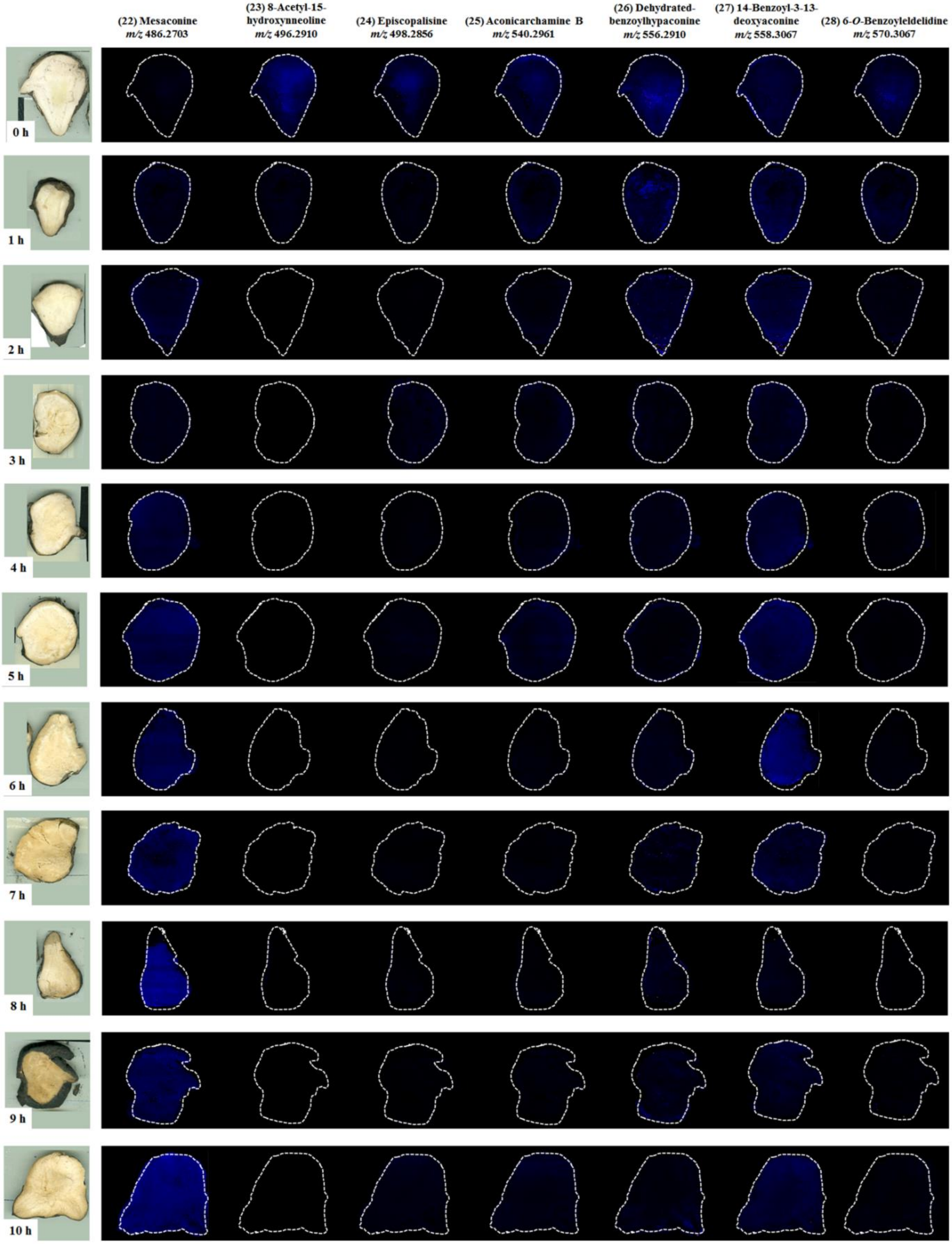
(22) Mesaconine
m/z 486.2703
(23) 8-Acetyl-15-hydroxynneoline
m/z 496.2910
(24) Episcopalisine
m/z 498.2856
(25) Aconicarchamine B
m/z 540.2961
(26) Dehydrated-benzoylhypaconine
m/z 556.2910
(27) 14-Benzoyl-3-13-deoxyaconine
m/z 558.3067
(28) 6-O-Benzoyleldelidine
m/z 570.3067
0 h
1 h
2 h
3 h
4 h
5 h
6 h
7 h
8 h
9 h
10 h

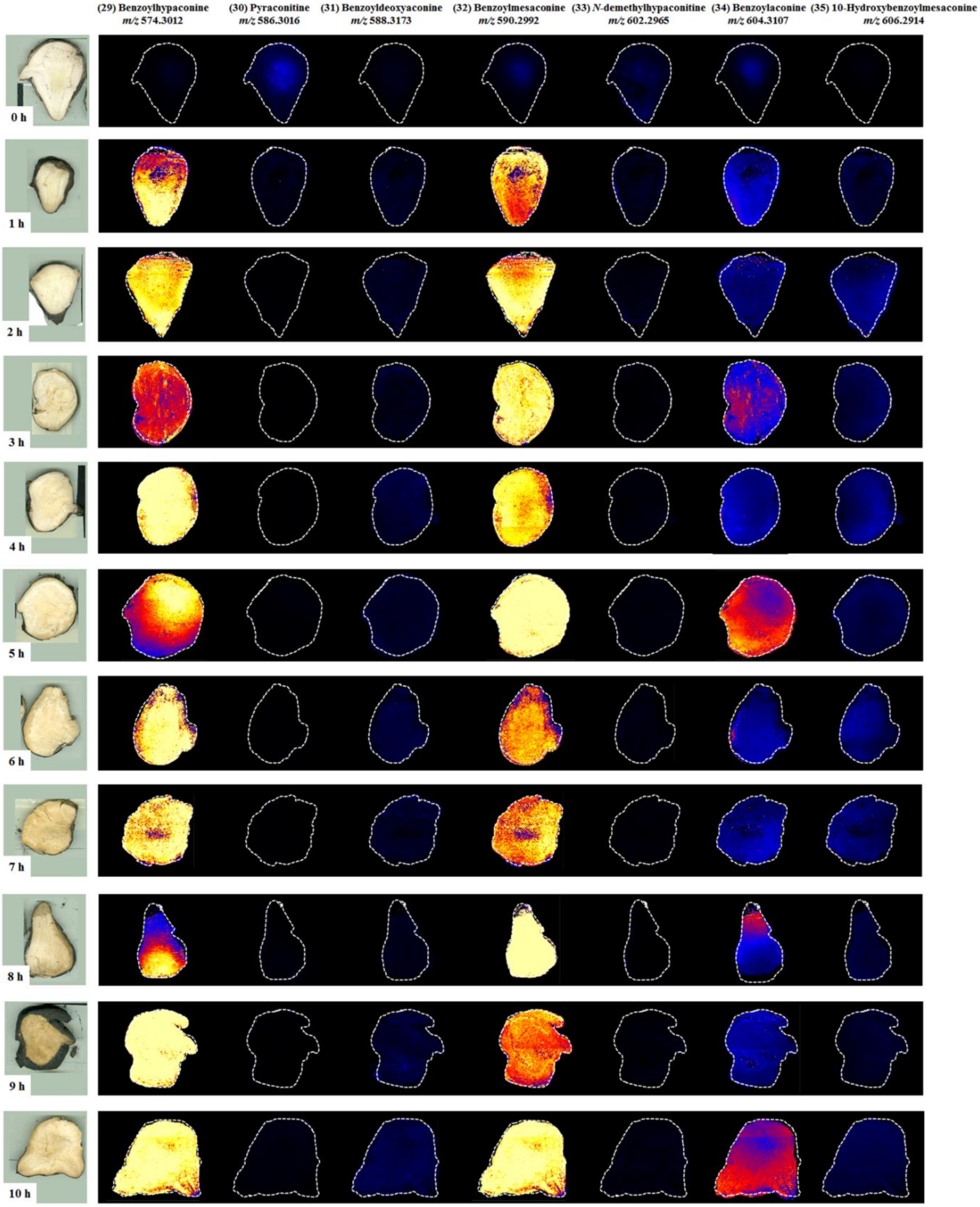

(29) Benzoylhypaconine
m/z 574.3012
(30) Pyraconitine
m/z 586.3016
(31) Benzoyldeoxyaconine
m/z 588.3173
(32) Benzoylmesaconine
m/z 590.2992
(33) N-demethylhypaconitine
m/z 602.2965
(34) Benzoylaconine
m/z 604.3107
(35) 10-Hydroxybenzoylmesaconine
m/z 606.2914
0 h
1 h
2 h
3 h
4 h
5 h
6 h
7 h
8 h
9 h
10 h

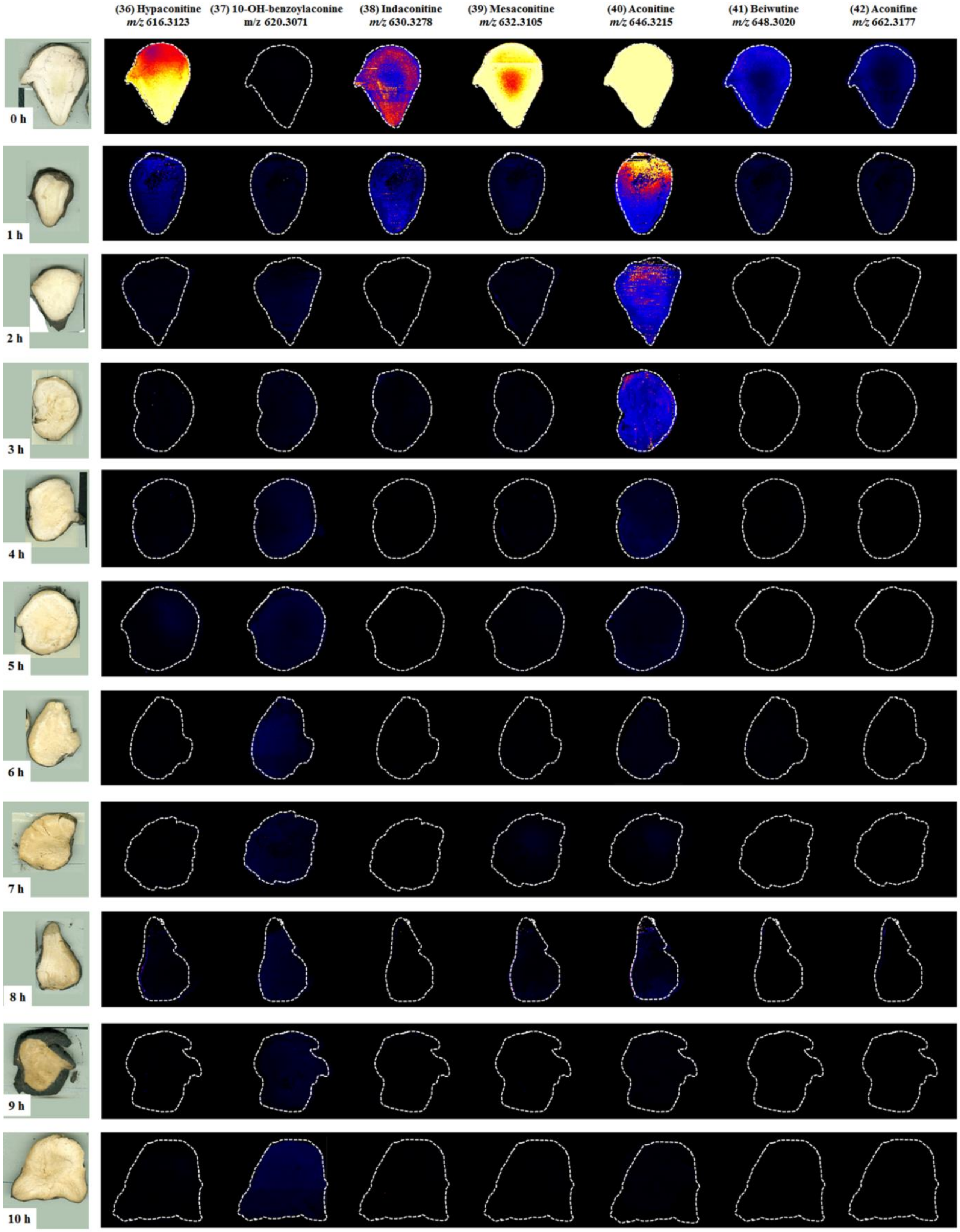

Supplementary Figure S3. DESI-MS images of 42 metabolic markers (**1-42**) in raw and processed Fuzi steamed for 0, 4.0 and 8.0 h.
