# Supplementary material for: Unveiling Dynamic Changes of Chemical Constituents in Raw and Processed Fuzi With Different Steaming Time Points Using Desorption Electrospray Ionization Mass Spectrometry Imaging Combined With Metabolomics
Source: Front Pharmacol. 2022 Mar 10;13:842890. doi: 10.3389/fphar.2022.842890 (PMC8960191; doi:10.3389/fphar.2022.842890)
Supplement: Supplementary file 4 [file Table2.docx]

Supplementary Material

**Table S2.** Recoveries of the standard addition test of the six ester-type alkaloids.

| **Compound** | **80% spiking** | | | **100% spiking** | | | **120% spiking** | | |
| --- | --- | --- | --- | --- | --- | --- | --- | --- | --- |
|  | **Found**  **(n=3, mg)** | **Spiked** | **Recovery (%)** | **Found**  **(n=3, mg)** | **Spiked** | **Recovery**  **(%)** | **Found**  **(n=3, mg)** | **Spiked** | **Recovery**  **(%)** |
| **32** | 0.320 | 0.257 | 98.93 | 0.317 | 0.321 | 97.94 | 0.318 | 0.385 | 100.83 |
| **34** | 0.075 | 0.060 | 98.06 | 0.075 | 0.075 | 97.88 | 0.075 | 0.090 | 100.59 |
| **29** | 0.135 | 0.109 | 98.19 | 0.135 | 0.136 | 97.86 | 0.135 | 0.163 | 101.04 |
| **22** | 0.065 | 0.052 | 99.30 | 0.065 | 0.065 | 97.14 | 0.065 | 0.078 | 100.38 |
| **40** | 0.501 | 0.400 | 98.73 | 0.498 | 0.500 | 98.41 | 0.501 | 0.600 | 100.67 |
| **36** | 0.037 | 0.032 | 98.65 | 0.037 | 0.040 | 97.01 | 0.037 | 0.048 | 100.28 |
